# Walking versus running and GFR trajectory in healthy young adults

**DOI:** 10.1371/journal.pone.0323392

**Published:** 2025-05-29

**Authors:** Ronit Calderon-Margalit, Ruth Lev Bar-Or, Arnon Afek, Dorit Tzur, Dana Levin, Dror Ben-Ruby, Ariel Furer, Gilad Twig, Karl Skorecki, Asaf Vivante

**Affiliations:** 1 Braun School of Public Health, Hadassah Medical Organization, Faculty of Medicine, Hebrew University, Jerusalem, Israel; 2 The Gertner Institute for Epidemiology and Health Policy Research, Sheba Medical Center, Ramat Gan, Israel; 3 The Dina Recanati School of Medicine, Reichman University, Herzliya, Israel; 4 Sheba Medical Center, Ramat Gan, Israel; 5 Faculty of Medical and Health Sciences, Tel-Aviv University, Tel-Aviv, Israel; 6 Department of Military Medicine, Faculty of Medicine, The Hebrew University of Jerusalem, Jerusalem, Israel; 7 The Institute of Endocrinology, Diabetes and Metabolism, Sheba Medical Center, Ramat Gan, Israel; 8 Department of Epidemiology and Preventive Medicine, School of Public Health, Faculty of Medical and Health Sciences, Tel-Aviv University, Tel-Aviv, Israel; 9 Incumbent of the Hella Gertner Chair for Research in Hypertension, Faculty of Medical and Health Sciences, Tel-Aviv University, Tel-Aviv, Israel; 10 Department of Nephrology, Rambam Health Care Campus, Rappaport Faculty of Medicine and Research Institute, The Technion–Israel Institute of Technology, Haifa, Israel; 11 Bar-Ilan University Faculty of Medicine, Safed, Israel,; 12 Pediatric Department B and Pediatric Nephrology Unit, Talpiot Medical Leadership Program, Edmond and Lily Safra Children’s Hospital, Faculty of Medical and Health Sciences Sheba Medical Center, Ramat Gan, Israel; Tribhuvan University Institute of Medicine, NEPAL

## Abstract

**Background:**

The effect of physical activity on the primary prevention of chronic kidney disease (CKD) is unclear. We assessed walking and running as exercise behaviors and their associations with individual-level risk for kidney function decline.

**Methods:**

We conducted a historical cohort study in which we followed 20,976 young adults. Participants were interviewed periodically about their lifestyle, and clinical parameters were assessed. The decline in estimated glomerular filtration rate (eGFR) over time was divided into quartiles. Using logistic regressions, we estimated the odds ratio (OR) for being in the slowest declining quartile by consistency of running or walking. We also used Cox proportional hazards models to estimate the associations of physical activity with future eGFR < 90 ml/min/1.73m^2^. All models were adjusted for age, sex, smoking status, family history of kidney diseases, BMI, blood-pressure, baseline eGFR and serum cholesterol.

**Results:**

During 9.5 years of follow-up, the eGFR decreased by 0.97 ml/min/1.73m^2^ per year. Participants who reported in two consecutive questionnaires on walking as a leisure time activity had an OR of 1.21 (95% confidence interval: 1.03–1.41) to have slow eGFR decline compared to those who were physically inactive. Participants who predominantly reported on running as their physical activity were less likely to be slow eGFR decliners (OR:0.81, 95% CI:0.71–0.93). Similarly, consistent walking was associated with decreased risk for future eGFR < 90 ml/min/1.73m^2^ in contrast to consistent running which was associated with an increased risk for reduced eGFR. All associations showed dose dependent effects in terms of the number of weekly activity sessions.

**Conclusions:**

Consistent walking, as opposed to consistent running, was associated with slower eGFR decline compared to inactive participants. These associations start already within the normal GFR range.

## Introduction

Chronic kidney disease (CKD) constitutes a major public health challenge, with an estimated 700–800 million patients globally and 2.6 million deaths in 2017 [1,2]. Therefore, primary prevention of CKD is a global priority. Inter-individual variability has been reported in the expected age-dependent decline of kidney function [3]. Several factors may contribute to this variability [4]. Lifestyle-related risk factors for kidney function decline are particularly worthy of investigation, as these are often modifiable and can be targeted as part of primary prevention interventions of progressive CKD.

Regular exercise behaviors may influence a person’s kidney function trajectory over time. However, evidence on physical exercise characteristics and kidney function trajectory or kidney disease progression in healthy individuals with normal kidney function is largely based on observational studies or cohorts which mainly investigated elderly patients [5] or persons with established CKD [4,6–8]. Moreover, while some studies suggest an association between physical activity as a kidney function protective behavior [9,10] others show no or inconsistent results [11,12]. Therefore, the long-term kidney health outcomes of consistent physical exercise as a lifestyle behavior among healthy young adults and its relation to other mediating factors remains largely unknown.

Consequently, we conducted a historical cohort study with the use of data from the Metabolic, Lifestyle, and Nutrition Assessment in Young Adults (MELANY) [13–15] Study of the Israel Defense Forces (IDF) Medical Corps and followed 20,976 apparently healthy young men and women to investigate the relationships between walking or running (the most prevalent leisure time physical activities globally [16]) and long-term risk of kidney function decline.

## Methods

We conducted a cohort study within the setting of the IDF Medical Corps Staff Periodic Examination Center (SPEC), utilizing data on military personnel who were initially examined between 01/01/1999 and 31/12/2010 and were followed until discharge from military service or 31/12/2020. Data was accessed for research purposes between 01/01/2021 and 31/12/2023.

### Study population and clinical assessment

**Fig 1:**
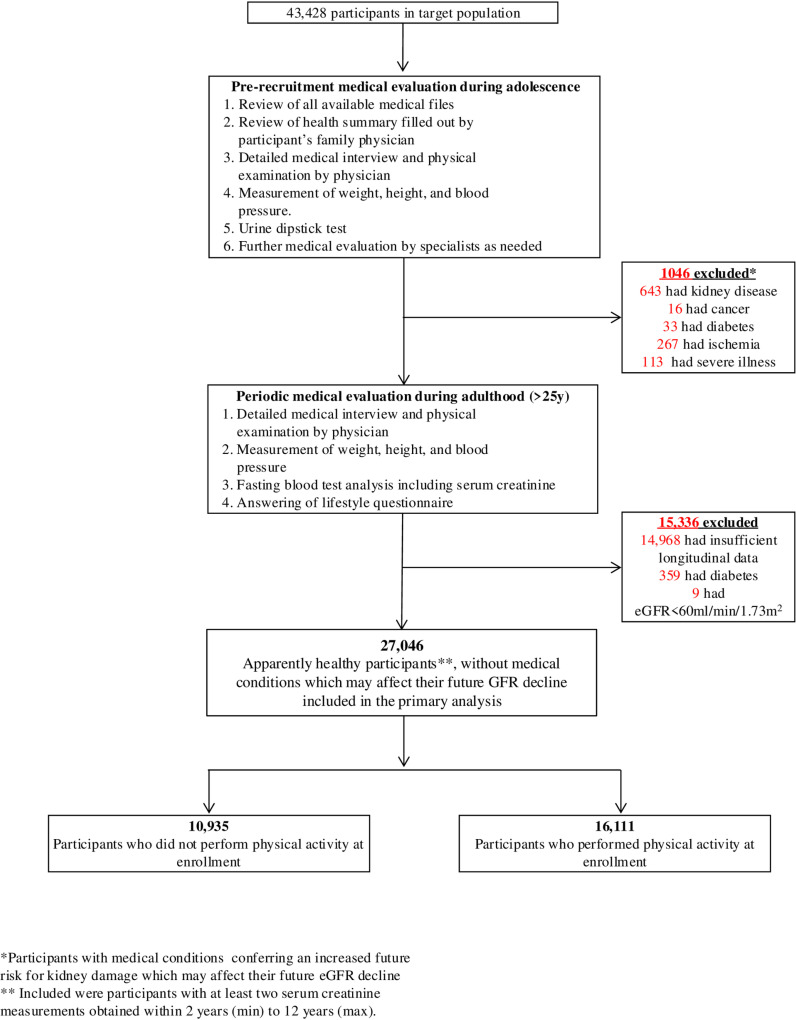
Assessment of study participants and designation of study groups.

The MELANY study [13–15] is an investigation of risk factors for common diseases in young adults, conducted at the IDF Medical Corps Staff Periodic Examination Center (SPEC). Since the mid-1990s, personnel remaining in the IDF beyond mandatory service were periodically evaluated (every 3–5 years) at the SPEC, beginning at approximately 25 years of age. At each examination, participants completed a detailed questionnaire to provide demographic, health, lifestyle and medical information. Blood samples were drawn following a 14-h fast. In addition, at each SPEC visit, weight, height, and blood pressure were measured, and complete medical interviews and physical examinations were performed. Between SPEC visits, primary care for IDF personnel was provided at designated clinics. For all participants, the SPEC database also includes information ascertained at age 17, coinciding with the initial military recruitment conscription assessment [17,18]. This medical evaluation includes a review of medical records obtained from primary care physicians, a detailed medical history and physical examination, including baseline measurements of weight and height and a manual sphygmomanometric blood pressure measurement (obtained on the right arm in the seated position), routine urinalysis, and, where indicated, referral for further specialty consultations as described below. Accordingly, for all cohort participants, the prior records were reviewed by IDF medical corps physicians, who then elicited and recorded a comprehensive medical history, and conducted a structured thorough and systematic physical examination, including anthropometric measurements, blood pressure, heart rate and a dipstick urinalysis test. If a specific diagnosis could not be fully verified or if its severity could not be graded after the primary medical evaluation, the participant was sent for additional tests and was referred to a board-certified specialist, who may order specific medical tests that will allow more precise diagnosis and classification. Moreover, for each diagnosis, including those established by the specialist during subsequent medical evaluation, the accuracy and completeness of the medical information was additionally assessed by two trained military service physicians who verified the medical information. Each diagnosis is assigned a numerical code and recorded in a central database. This process is uniform for the entire cohort (Fig 1).

### Inclusion and exclusion criteria

The study comprised 20,976 participants with at least two serum creatinine measurements and at least two SPEC visits. We included persons with at least two serum creatinine measurements obtained over a time interval of between 2–12 years. These participants appeared similar to those with fewer than two serum creatinine measurements in the target population (Table S1). Patients were excluded from the study if they had any of the following at the time of enrollment: (a) confirmed type 1 or type 2 diabetes; (b) estimated glomerular filtration rates (eGFR) of less than 60 ml/min/1.73m^2^; (c) current or past medical history of any stage of acute or chronic kidney disease; (d) microscopic hematuria or proteinuria; (e) ischemic heart disease; (f) past or current diagnosis of cancer and (g) major chronic illness preventing them from military duty (Fig 1).

### Physical activity assessment

At each SPEC examination, participants completed a detailed questionnaire containing structured multiple choice questions to provide demographic, health, lifestyle (including smoking), and medical information (including family history of kidney disease). Leisure-time physical activity was assessed in terms of type of exercise (e.g., running, walking, cycling, etc.), weekly frequency, and duration (<45 minutes vs. ≥ 45 minutes per session).

### Follow-up and outcomes

Participants were followed-up from their first SPEC visits (01/01/1999-31/12/2010), until retirement from service, or 31/12/2020, whichever came first. We delineated two study outcomes: annual rate of eGFR decline and eGFR < 90 ml/min/1.73m^2^. eGFR was calculated using the Chronic Kidney Disease Epidemiology Collaboration (CKD-EPI) 2021 equation [19]. Creatinine measurements before 2010 were corrected by 4% reductions, to account for the transition from the Jaffe method to enzymatic creatinine measurements in Israel in 2010 [20]. The primary outcome of mean annual changes in eGFR trajectory was calculated as the eGFR difference divided by the interval in number of years between measurements and then categorized into quartiles. We looked at the lowest (slowest decline) quartile compared to the three upper quartiles.

### Statistical analysis

Descriptive statistics of the study population by physical activity are presented as means with standard deviation or proportions. We studied consistency in performing specific types of physical activity, looking into whether a reported activity in the first questionnaire was reported also in a subsequent questionnaire. The associations between consistency of performing a specific type of physical activity and the lowest quartile of eGFR decline were estimated using logistic regressions where inconsistent and consistent physical activity were compared to no physical activity at baseline, controlling for sex, age, smoking, BMI, baseline eGFR, LDL-cholesterol, hypertension, and family history of kidney disease (all from the first questionnaire). We further studied the associations with the weekly mean number of activity sessions (averaged across the two questionnaires) and with the duration of sessions in each questionnaire (answers could not be averaged).

We further estimated the association between types of physical activity and reaching eGFR below 90 ml/min/1.73m^2^, counting time from first eGFR assessment in the cohort until reaching an eGFR < 90 ml/min/1.73m^2^ or last eGFR assessment. Associations were estimated using Cox proportional hazards models. All analyses were conducted in R, version 3.6.1, with the survival models fitted with the coxph function of the survival package. Two-sided p values of less than 0.05 were considered statistically significant.

The Institutional Review Board of the Israel Defense Forces Medical Corps approved the study and waived the requirement for written informed consent on the basis of preserving participants’ anonymity. More specifically, data was analyzed at secured computer stations in the IDF and in an anonymized fashion, such that the data analysts could not track back the participants.

## Results

### Characteristics of study participants

The characteristics of the cohort of 20,976 participants (85% men) with stratification according to leisure time physical activity (any vs. none) at enrollment are presented in Table 1. The mean age at the prerecruitment medical assessment was 17.5 ± 1.4 years and the mean BMI was 21.1 ± 3.3 kg/m^2^. The mean systolic and diastolic BP were 118.3 ± 11.4 and 73.6 ± 9.1 mmHg respectively.

**Table 1:** Characteristics of the study population according to physical activity at enrollment*.

	All participants (N = 20,976)	Participants who did not perform physical activity (N = 7,464)	Participants who performed physical activity at enrollment (N = 13,512)
Gender			
Male	18,206 (86.8%)	6,323 (84.7%)	11,883 (87.9%)
Female	2,770 (13.2%)	1,141 (15.3%)	1,629 (12.1%)
Education			
Less than 12 years	5,002 (23.8%)	1,991 (26.7%)	3,011 (22.2%)
12 years or above	6,707 (23.6%)	2,681 (35.9%)	4,026 (29.8%)
Bachelor’s degree	6,363 (32.0%)	2,028 (27.2%)	4,335 (32.1%)
Master’s degree/PhD	2,904 (13.8%)	764 (10.2%)	2,140 (15.8%)
Blood pressure (mmHg)			
Diastolic	73.6 ± 9.1	73.6 ± 9.2	73.6 ± 9.0
Systolic	118.3 ± 11.4	118.4 ± 11.5	118.3 ± 11.4
**Measurements in adulthood**			
Age (years)	32.4 ± 5.4	32.7 ± 5.5	32.1 ± 5.3
BMI (kg/m^2^)	25.8 ± 4.2	25.8 ± 4.5	25.7 ± 4.0
Current smoker	5,578 (26.6%)	2,375 (31.8%)	3,203 (23.7%)
Blood pressure (mm Hg)			
Diastolic	73.7 ± 9.0	73.7 ± 9.2	80.0 ± 8.9
Systolic	117.9 ± 11.4	117.8 ± 11.3	117.9 ± 11.4
Hypertension^#^ N (%)	582 (2.8%)	242 (3.2%)	340 (2.5%)
Hyperlipidemia^^^ N (%)	2,259 (10.9%)	937 (12.8%)	1,322 (9.9%)
First eGFR (ml/min/1.73m^2^)	115.5 ± 10.2	116.0 ± 10.0	115.2 ± 10.3
First creatinine measurement (mg/dl)	0.99 ± 0.15	0.97 ± 0.16	1.0 ± 0.15
Time span between first and last creatinine measurement^**^	8.8 ± 3.0	8.4 ± 3.1	9.0 ± 2.9
Annual eGFR change (ml/min/1.73m^2^ per year)	-0.97 ± 1.6	-0.95 ± 1.7	-0.98 ± 1.5
Number of lifestyle questionnaires	3.0 ± 0.94	2.9 ± 0.86	3.0 ± 0.96
Calendar year of first questionnaire, N (%)			
1999-2004	12,121 (57.8%)	4,231 (56.7%)	7,890 (58.4%)
2005-2009	7,343 (35.0%)	2,617 (35.1%)	4,726 (35.0%)
2010 +	1,512 (7.2)	616 (8.3%)	896 (5.8%)
Time span (years) between first and last lifestyle questionnaire	8.7 ± 3.9	8.0 ± 3.8	9.1 ± 3.9

* Values are means ± SD.

** Included were all subjects that have at least two creatinine measurements within of 2–12 years from the first measurement. ^#^ Hypertension was defined as systolic BP above 140 mmHg or diastolic BP above 90 mmHg which was obtained on several occasions. ^^^Hyperlipidemia was defined as fasting serum LDL level above 160 mg/dl. First lifestyle questionnaire was chosen to be the earliest questionnaire which included creatinine measurements ± 1 yr.

Participants were 32.4 ± 5.4 years old at their first SPEC visit and their BMI was 25.8 ± 4.2 kg/m^2^. Participants averaged three SPEC visits per person during follow up (SD: 0.94, range 2–7), and their average first eGFR measured 115.6 (±10.0) ml/min/1.73m^2^ for men and 115.0 (±11.6) ml/min/1.73m^2^ for women. The annual average eGFR declined by 0.97 (±1.6) ml/min/1.73m^2^ per year. Mean quartiles of eGFR reduction rates were 0.31 ml/min/1.73m^2^ per year or less for the bottom quartile (Q1 = “slow GFR decline”) to 1.61 ml/min/1.73m^2^ per year or more for the top quartile (Q4 = “fast GFR decline”). The first estimated eGFR measurement at enrollment for slow decliners (Q1) and fast decliners (Q4) were 108.2 ± 10.8 and 117.3 ± 9.12 ml/min/1.73m^2^, respectively (p < 0.001).

Among the 13,512 participants who reported engaging in physical activity at enrollment, the two most common types of activity were running and walking, which were documented for 26.3% and 18.7% participants respectively. These activities were followed by team sports (9.8%), individual gym fitness workouts (9.2%) and cycling (7.4%). Other activities were reported by less than 5% of the study participants. Given that walking and running were the most common physical activities employed, our primary analysis focused on the associations of consistent walking or running as habitual exercise activities and the eGFR trajectory.

### Walking versus running and GFR trajectory

Table 2 presents the associations between consistent and inconsistent engagement in running or walking compared to inactive participants and shows the odds for having a trajectory of slow eGFR decline (Q1). In multivariable models we found that consistent walking showed the highest odds for slow GFR decline as compared to inconsistent or inactive participants. Participants who reported in two consecutive questionnaires that they engaged in walking had an OR of 1.21 (95% CI:1.03–1.41) for being in the slowest eGFR decline quartile (Q1), whereas those who were not consistent in walking had an insignificant increased OR of 1.06 (0.81–1.22). Conversely, participants who consistently engaged in running had significantly decreased odds for slow eGFR decline (OR:0.81, 95% CI:0.71–0.93), whereas inconsistent running was associated with an insignificant reduction in odds (OR:0.87, 95% CI: 0.74–1.01). Similar results were obtained when the first and last questionnaires were analyzed (Table 2). Among consistent walkers, the average number of sessions per week was significantly associated with slow eGFR decline (OR:1.06 [1.00–1.12] per session per week), whereas the number of weekly running sessions was associated with reduced odds of being slow decliners (OR: 0.93 [0.90–0.98] per session per week). Supplementary figure 1 (Fig S1) presents the mean eGFR by age and type of physical activity.

**Table 2:** The association between consistent habitual running or walking and a slow trajectory of estimated GFR decline (quartile 1)*.

		Walking		Running	
Questionnaires of interest	Physical activity level	OR (95% CI)	N	OR (95% CI)	N
**First and second**	Never engaged in physical activity	1.00 **(ref)**	6,302	1.00 **(ref)**	6,302
	Consistent physical activity (running or walking) in first and second questionnaire	1.21 (1.03-1.41)	1,163	0.81 (0.71-0.93)	2,108
	Mixed: Didn’t persist in second questionnaire	1.06 (0.81-1.22)	1,368	0.87 (0.74-1.01)	1,453
**First and last**	Never engaged in physical activity	1.00 (reference)	6,302	1.00 (reference)	6,302
	Consistent physical activity (running or walking) in first and last questionnaire	1.23 (1.04-1.45)	987	0.81 (0.71-0.93)	2,042
	Mixed: Didn’t persist in second questionnaire	1.05 (0.91-1.22)	1,544	0.87 (0.75-1.01)	1,519

* Models are adjusted for sex, BMI, baseline eGFR, LDL-cholesterol, hypertension, age, smoking, family history of kidney disease (all at first questionnaire), number of questionnaires.

We further assessed whether any of these physical activities reduced future risks of reaching an estimated GFR below 90 ml/min/1.73m^2^ over the study period (Table 3). During 200,005 person years of follow-up (mean: 9.53 ± 4.8 years), 5,409 (25.8%) participants reached an eGFR below 90 ml/min/1.73m^2^. In multivariate Cox models consistent walking was associated with reduced risk for future GFR below 90 ml/min/1.73m^2^ (HR of 0.82 (0.72–0.95)), whereas inconsistent walking was not associated with a reduced risk (HR: 0.94, 95% CI: 0.82–1.07). Conversely, consistent running was associated with an increased risk for future GFR below 90 ml/min/1.73m^2^ (HR: 1.22, 95% CI: 1.09–1.35) while inconsistent running was not associated with increased risk (HR: 1.07, 95% CI: 0.93–1.20). Studying the association of the frequency of physical activity and future eGFR below 90 ml/min/1.73m^2^, we found that walking had an HR of 0.95 (0.90–1.00) for each additional practice per week and running had an HR of 1.07 (1.03–1.11) per practice per week. These associations were not modified by the duration of each practice. Restricting the analyses to those who had less than five years between the first and second questionnaires did not materially change the results. Similarly, stratifying the analyses of the SPEC by year of first SPEC visit (before or after 2010) showed similar results (Table S2). Furthermore, analysis by sex failed to find significant association in females, given the small number of female participants (Table S3).

**Table 3:** Association between consistent habitual walking or running and future risk of estimated GFR below 90 ml/min/1.73m^2^.

	Walking		Running	
Physical activity level	N	HR (95% CI)	N	HR (95% CI)
Never engaged in physical activity	6,302	1 (ref)	6,302	1 (ref)
Consistent: Answered “walking”/”running” in first and second questionnaire	1,163	0.82 (0.72-0.95)	2,108	1.22 (1.09-1.35)
Mixed: Didn’t persist in second questionnaire	1,368	0.94 (0.82-1.07)	1,453	1.07 (0.93-1.20)

Models controlled for age, sex, BMI, baseline eGFR, LDL-cholesterol, hypertension, smoking, family history of kidney disease (all at first questionnaire).

## Discussion

In this study of 20,976 apparently healthy young adults, we found that physical activity behavior of consistent walking, but not consistent running, was associated with slower longitudinal eGFR decline compared to inactive participants. This association starts already within the normal GFR range and was independent of other established risk factors for kidney function impairment. Similar to previous observations [21], we showed that the yearly average physiological GFR decline during adulthood among healthy persons is ~ 1 ml/min/1.73m^2^. In this respect, walking was found to be associated with a higher likelihood of being at the slower eGFR trajectory decline quartile. In addition, walking was associated with lower likelihoods of reaching a eGFR below 90 ml/min/1.73m^2^ at the end of follow-up. Conversely, we found that habitual running might increase the physiological age-dependent kidney function decline. Our findings suggest that among young adults with normal kidney function, habitual walking may have a kidney protective effect.

Several limitations of this study warrant consideration. First, physical activity characteristics (type, duration and frequency) were self-reported and not directly or objectively measured. Nonetheless, we focused on consistent reports across at least two structured questionnaires taken on different occasions and over several years. Second, eGFR was estimated based on serum creatinine and was not measured. This does not account for diet, hydration, or chronic NSAIDs use, which we did not have data on. In addition, we did not measure cystatin C. However, in the normal GFR range there is strong correlation between measured and estimated GFR using the CKD-EPI 2021 formulation [19]. Third, our study population included mainly young and healthy men, limiting the generalizability of our findings to other populations, or even having meaningful insights on gender differences. Finally, we did not have comprehensive information regarding participants’ muscle mass, nutrition and diet, which can also affect kidney health.

The strengths of this study include uniform and detailed data from periodic clinical assessments as well as systematic follow-ups and outcome definitions. In addition, the use of measured (rather than reported) values for the body mass index, blood pressure, and laboratory results with direct measurements of serum creatinine, lipids and glucose in fresh venous blood, provided reliable determinations. This allowed us to control for other confounding CKD risk factors. Moreover, the medical assessments of all study participants during adolescence which were performed under similar protocols as well as subsequent standardized follow-up medical evaluations, allowed us to adhere to consistent inclusion and exclusion criteria and to uniquely study the question of the association between physical activity among healthy adults and kidney function decline in our cohort.

Few previous studies [9,22] have shown an association between physical activity and improved kidney function. In a short-term prospective study among elderly participants (age 70–89 years) with baseline eGFR of 54 ml/min/1.73m^2^, moderate-intensity physical activity slowed the rate of decline in eGFR over a subsequent follow-up period of two years [22]. In another large cohort study of nearly 200,000 Taiwanese adults, a higher level of physical activity was associated with smaller decreases in eGFR and in a lower risk of developing CKD [9]. In a recent comprehensive study which included two large and heterogeneous cohorts from the UK Biobank, with participants having a mean age of 63 years, physical activity of light, moderate or vigorous intensity was associated with lower risks of developing CKD [5]. The latter association of intense physical activity as a kidney protective effect is opposed to our findings as well as to the conventional physiological knowledge and previous studies [23]. Our study confirms the protective effect of walking as opposed to running among healthy individuals already within the normal GFR range.

The lack of a renal protective effect among participants who run may have several explanations. First, during running, blood pressure increases [24,25], leading to renal autoregulation adjustments which in the long-term may affect the overall GFR trajectory. Second, runners may have subclinical myoglobinuria and hemoglobinuria which may lead to subtle kidney damage effecting the overall long-term physiological GFR decline [26]. Third, runners, as opposed to walkers, are prone to recurrent small volume depletion events, as well as to chronic use of non-steroidal anti-inflammatory (NSAIDs) nephrotoxic medications, both of which can contribute to ongoing subclinical kidney damage [27–29]. Finally, it is plausible that this negative effect of running on GFR trajectory is an isolated kidney-specific phenomenon [28,30], otherwise showing protective effect on cardiovascular morbidity and mortality. Therefore, the association between running and physiological GFR decline needs further investigation.

Another interesting finding gleaned from our study is that participant with GFR towards the higher limit of the normal GFR range, had a faster GFR decline compared to participants with lower, normal values. While no single definition of glomerular hyperfiltration has been agreed upon, a commonly used definition for normal GFR is 90–120 ml/min/1.73m^2^ [31–34]. In this study, participants with GFR at the ~ 110 ml/min/1.73m^2^ range had a slower GFR decline compared with those in the 120 ml/min/1.73m^2^ range. This may imply that a GFR of 120 ml/min/1.73m^2^ represents relative “hyperfiltration” which may be more detrimental to the future kidney function trajectory over time and, therefore, we should set the “normal” GFR at the 110 ml/min/1.73m^2^ range. Consequently, this highest eGFR level within the normal GFR range may constitute an independent risk factor for future faster GFR decline among healthy adults.

In conclusion, this large-scale, long-term follow-up study suggests that consistent walking as a physical exercise has a protective effect on kidney health that may delay the physiological kidney function decline. This is an important finding given that mitigating age-related reduction in GFR by habitual walking behavior can be easily applied and prescribed for healthy individuals, as well as to persons with increased risk for future chronic kidney disease.

## Supporting information

Table S1 Characteristics of 21,038 apparently healthy participants who were not included in the primary analysis compared to the study cohort. (DOCX)

Table S2 Sensitivity analysis by year of first SPEC visit. (DOCX)

Table S3 Association between consistent habitual running or walking and a slow trajectory of estimated GFR decline stratified by sex. (DOCX)

Fig S1 eGFR trajectories by type of physical activity. (TIF)
