# Supplementary material for: Walking versus running and GFR trajectory in healthy young adults
Source: PLoS One. 2025 May 29;20(5):e0323392. doi: 10.1371/journal.pone.0323392 (PMC12121832; doi:10.1371/journal.pone.0323392)
Supplement: Table S1 — (DOCX) [file pone.0323392.s001.docx]

| **Supplementary Table 1**. **Characteristics of 21,038 apparently healthy participants who were not included in the primary analysis compared to the study cohort** | | |
| --- | --- | --- |
| **Characteristic** | **Study Cohort Participants** | **Excluded Participants** |
| Males, n (%) | 18,206 (86.8%) | 17,808 (84.6%) |
| Females, n (%) | 2,770 (13.2%) | 3,230 (15.3%) |
| BMI (at first lifestyle question), (kg/m^2^) | 25.8 ± 4.2 | 25.8 ± 4.5 |
| First eGFR measure, (ml/min/1.73m^2^) | 115.5 ±10.2 | 114.3 ±10.9 |
| First eGFR measure - Males | 115.6 ± 10.0 | 114.2 ± 10.7 |
| First eGFR measure - Females | 115.0 ± 11.6 | 114.6 ± 12.1 |
| **Comorbidities** | | |
| Hypertension, n (%) | 582 (2.8%) | 705 (3.3%) |
| Hyperlipidemia, n (%) | 2,259 (10.9%) | 2,219 (10.5%) |
| **Current Smoker** | | |
| “No”, n (%) | 15,392 (73.4%) | 15,377 (73.1%) |
| “Yes”, n (%) | 5,578 (26.6%) | 5,658 (26.9%) |
| **Blood pressure (mmHg) during adulthood** | | |
| Diastolic pressure | 73.6 ± 9.1 | 73.8 ± 9.2 |
| Systolic pressure | 118.3 ± 11.4 | 119.5 ± 11.7 |
| **Blood pressure (mmHg) at adolescence** | | |
| Diastolic pressure | 73.7 ± 9.0 | 72.7 ± 8.1 |
| Systolic pressure | 117.9 ± 11.4 | 118.1 ± 12.0 |
