# Supplementary material for: Walking versus running and GFR trajectory in healthy young adults
Source: PLoS One. 2025 May 29;20(5):e0323392. doi: 10.1371/journal.pone.0323392 (PMC12121832; doi:10.1371/journal.pone.0323392)
Supplement: Table S2 — (DOCX) [file pone.0323392.s002.docx]

| **Supplementary Table 2. Sensitivity analysis by year of first SPEC visit** | | | |
| --- | --- | --- | --- |
| **Walking** | | | |
|  | **Overall** | **<2010**  **N=8188** | **>2010**  **N=645** |
| **Outcome: Quartile of slowest eGFR decline** | **OR (95% CI)** | **OR (95% CI)** | **OR (95% CI)** |
| Reference level: never engaged in physical activity | 1.00 (ref) | 1.00 (ref) | 1.00 (ref) |
| Persisting: answered “walking” in first and second questionnaire | 1.21 (1.03-1.41) | 1.17 (0.99-1.37) | 2.13 (1.15-4.64) |
| Mixed- didn’t persist in second questionnaire | 1.06 (0.81-1.22) | 1.01 (0.86-1.18) | 2.00 (1.13-3.51) |
| **Outcome: eGFR<90 ml/min/1.73m^2^** | **HR (95% CI)** | **HR (95% CI)** | **HR (95% CI)** |
| Reference level: never engaged in physical activity | 1 (ref) |  | - |
| Persisting: answered “walking” in first and second questionnaire | 0.82 (0.72-0.95) | 0.84 (0.73-0.98) | 0.40 (0.15-1.05) |
| Mixed - didn’t persist in second questionnaire | 0.94 (0.82-1.07) | 0.92 (0.81-1.06) | 1.32 (0.70-2.50) |
| **Running** | | | |
|  | **Overall** | **<2010**  **N=8993** | **>2010**  **N=870** |
| **Outcome: Quartile of slowest decline** | **OR (95% CI)** | **OR (95% CI)** | **OR (95% CI)** |
| Reference level: never engaged in physical activity | 1.00 (ref) | 1.00 (ref) | 1.00 (ref) |
| Persisting: answered “running” in first and second questionnaire | 0.81 (0.71-0.93) | 0.80 (0.70-0.93) | 0.97 (0.63-1.50) |
| Mixed - didn’t persist in second questionnaire | 0.87 (0.74-1.01) | 0.84 (0.71-0.99) | 1.26 (0.76-2.09) |
| **Outcome: eGFR<90 ml/min/1.73m^2^** | **HR (95% CI)** | **HR (95% CI)** | **HR (95% CI)** |
| Reference level: never engaged in physical activity | 1 (ref) | 1.00 (ref) | 1.00 (ref) |
| Persisting: answered “running” in first and second questionnaire | 1.22 (1.09-1.35) | 1.20 (1.08-1.33) | 1.17 (0.73-1.88) |
| Mixed - didn’t persist in second questionnaire | 1.07 (0.93-1.20) | 1.05 (0.92-1.19) | 1.24 (0.67-2.27) |
