# Supplementary material for: Walking versus running and GFR trajectory in healthy young adults
Source: PLoS One. 2025 May 29;20(5):e0323392. doi: 10.1371/journal.pone.0323392 (PMC12121832; doi:10.1371/journal.pone.0323392)
Supplement: Table S3 — (DOCX) [file pone.0323392.s003.docx]

| **Supplementary Table 3: The association between consistent habitual running or walking and a slow trajectory of estimated GFR decline (quartile 1) stratified by sex** | | | | | |
| --- | --- | --- | --- | --- | --- |
| **Males** | | | | | |
|  |  | **Walking** | | **Running** | |
| **Questionnaires of interest** | **Physical activity level** | **OR (95% CI)** | **N** | **OR (95% CI)** | **N** |
| **First and second** | Never engaged in physical activity | 1.00 **(ref)** | 5,351 | 1.00 **(ref)** | 5,351 |
|  | Consistent physical activity (running or walking) in first and second questionnaire | 1.22 (1.06-1.46) | 966 | 0.83 (0.72-0.95) | 2,050 |
|  | Mixed: Didn’t persist in second questionnaire | 1.06 (0.90-1.28) | 1,114 | 0.90 (0.77-1.05) | 1,386 |
| **First and last** | Never engaged in physical activity | 1.00 (reference) | 5,351 | 1.00 (reference) | 5,351 |
|  | Consistent physical activity (running or walking) in first and last questionnaire | 1.24 (1.03-1.50) | 816 | 0.83 (0.72-0.95) | 1,985 |
|  | Mixed: Didn’t persist in second questionnaire | 1.06 (0.91-1.25) | 1,264 | 0.90 (0.77-1.06) | 1,451 |
| **Females** | | | | | |
|  |  | **Walking** | | **Running** | |
| **Questionnaires of interest** | **Physical activity level** | **OR (95% CI)** | **N** | **OR (95% CI)** | **N** |
| **First and second** | Never engaged in physical activity | 1.00 **(ref)** | 951 | 1.00 **(ref)** | 951 |
|  | Consistent physical activity (running or walking) in first and second questionnaire | 1.16 (0.80-1.70) | 197 | 0.66 (0.40-1.30) | 58 |
|  | Mixed: Didn’t persist in second questionnaire | 1.00 (0.70-1.40) | 254 | 0.52 (0.30-1.00) | 67 |
| **First and last** | Never engaged in physical activity | 1.00 (reference) | 951 | 1.00 (reference) | 951 |
|  | Consistent physical activity (running or walking) in first and last questionnaire | 1.19 (0.80-1.77) | 171 | 0.66 (0.34-1.31) | 57 |
|  | Mixed: Didn’t persist in second questionnaire | 0.99 (0.70-1.38) | 280 | 0.53 (0.28-0.99) | 68 |
