# Supplementary figures and images for: Walking versus running and GFR trajectory in healthy young adults
Source: PLoS One. 2025 May 29;20(5):e0323392. doi: 10.1371/journal.pone.0323392 (PMC12121832; doi:10.1371/journal.pone.0323392)

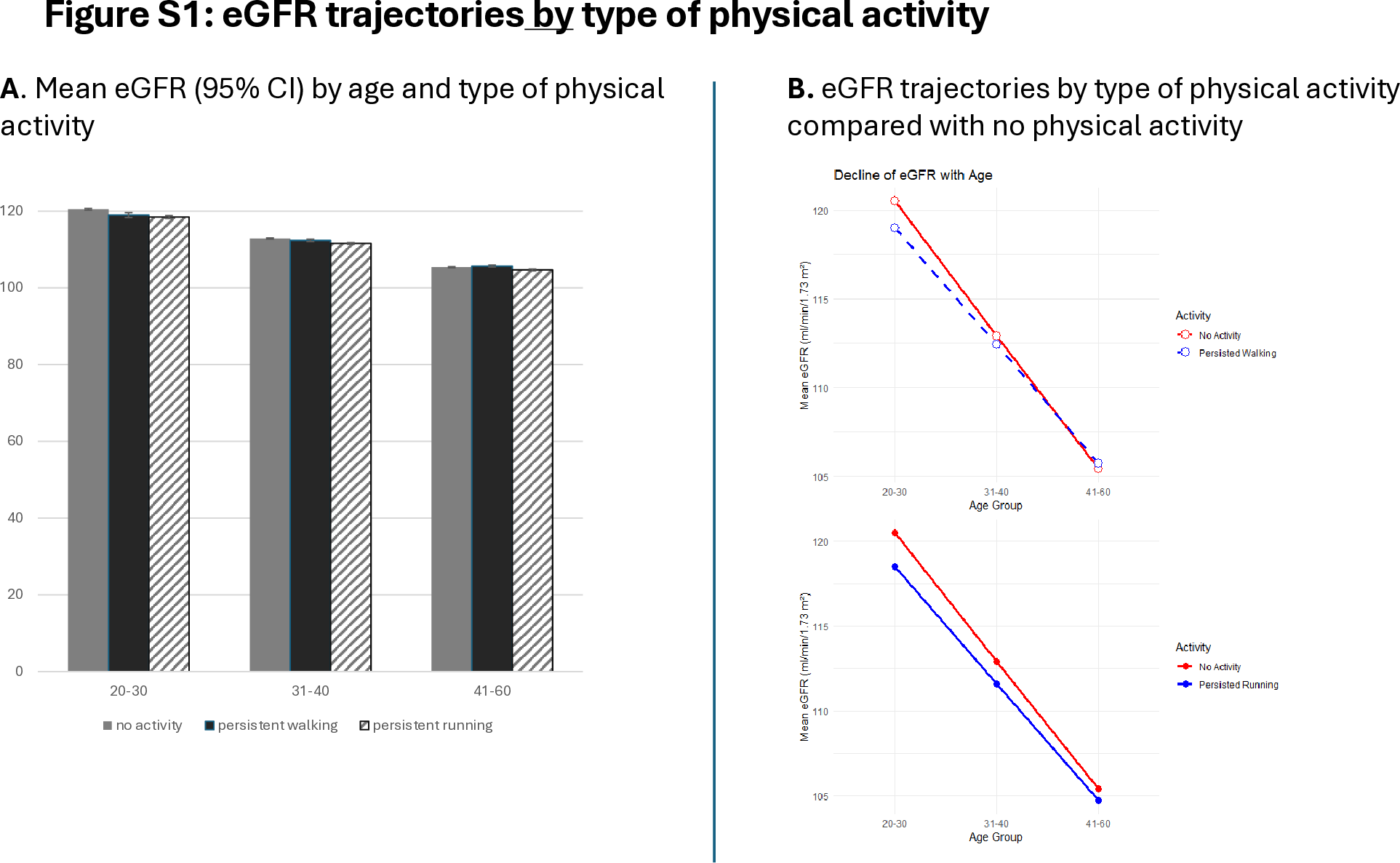

Supplement: Fig S1 — (TIF) [file pone.0323392.s004.tif]
